# Factors associated with clinical nurse’s mental health: a qualitative study applying the social ecological model

**DOI:** 10.1186/s12912-024-02005-9

**Published:** 2024-05-16

**Authors:** Qiang Yu, Chongmei Huang, Yusheng Tian, Jiaxin Yang, Xuting Li, Meng Ning, Zengyu Chen, Jie Du, Jiaqing He, Yamin Li

**Affiliations:** 1 Clinical Nursing Teaching and Research Section, The Second Xiangya Hospital, Central South University, Changsha, China; 2 School of Nursing, Ningxia Medical University, Yinchuan, China; 3 School of Nursing, Changsha Medical University, Changsha, China; 4 Xiangya School of Nursing, Central South University, Changsha, China

**Keywords:** Clinical nurses, Mental health, Social ecological model, Focus group, China

## Abstract

**Background:**

The prevalence of burnout, depression, and anxiety among Chinese nurses was 34%, 55.5%, and 41.8% respectively. Mental health problems have significant impacts on their personal well-being, work performance, patient care quality, and the overall healthcare system. Mental health is influenced by factors at multiple levels and their interactions.

**Methods:**

This was a descriptive qualitative study using phenomenological approach. We recruited a total of 48 nurses from a tertiary hospital in Changsha, Hunan Province, China. Data were collected through focus group interviews. Audio-recorded data were transcribed and inductively analysed.

**Results:**

Four major themes with 13 subthemes were identified according to the social ecological model: (1) individual-level factors, including personality traits, sleep quality, workplace adaptability, and years of work experience; (2) interpersonal-level factors, encompassing interpersonal support and role conflict; (3) organization-level factors, such as organizational climate, organizational support, career plateau, and job control; and (4) social-level factors, which included compensation packages, social status, and legislative provision and policy.

**Conclusions:**

Our study provides a nuanced understanding of the multifaceted factors influencing nurses’ mental health. Recognizing the interconnectedness of individual, interpersonal, organizational, and social elements is essential for developing targeted interventions and comprehensive strategies to promote and safeguard the mental well-being of nurses in clinical settings.

**Trial and protocol registration:**

The larger study was registered with Chinese Clinical Trial Registry: ChiCTR2300072142 (05/06/2023) https://www.chictr.org.cn/showproj.html?proj=192676.

**Reporting method:**

This study is reported according to the Consolidated Criteria for Reporting Qualitative Research (COREQ).

## Background

The prevalence of mental health problem among clinical nurses is high. As the largest group of health systems, clinical nurses play a crucial role in promoting health and preventing disease [1]. Although they are trained to provide care for their patients, they rarely cared about themselves [1]. Clinical nurses are suffering from mental health problems, including stress, anxiety, depression, and burnout. A meta-analysis involving 45,539 nurses from 49 countries revealed that a global prevalence of burnout symptoms was 11.23% across various specialties [2]. In Australia, the prevalence of depression, anxiety and stress among nurses was 32.4%, 41.2% and 41.2%, respectively [1]. In Italy, the prevalence of generalized anxiety disorder among nurses is 50% [3].In Spain, 68% of nurses had depression, anxiety, insomnia and distress to some degree, and 38% of them had moderate or severe symptoms [4]. A survey of clinical nurses from 30 Chinese provinces indicated that the rates of burnout, depression, and anxiety was 34%, 55.5%, and 41.8%, respectively [5]. Mental health problems may compromise physical, mental, and social health and even increase suicide risk [6].

The mental health problems among clinical nurses affects their personal well-being, work performance, patient care quality, and the healthcare system. Remarkably, their mental health problems not only heighten the risk of physical conditions such as heart disease, chronic pain, gastrointestinal distress, and even mortality [7], but also correlate with absenteeism, intention to leave, and elevated turnover rates [8, 9]. These increased turnover rates exacerbate the financial challenges faced by healthcare institutions [10]. The presence of one or more of these mental health problems can contribute to occupational mishaps, including medical errors [1, 11], compromised work performance, and a pessimistic workplace demeanor [12]. Nurses with mental health problems are at 26–71% more likely to make medical errors [13]. Furthermore, their mental health may imperil the well-being of patients and the quality of health services [14]. Moreover, these challenges can contribute to reputation harm, diminished productivity, and decreased clinical efficacy of the hospital [15]. Therefore, it is necessary to identify factors associated with their mental health for developing and implementing targeted intervention.

Previous studies have identified several factors associated with clinical nurses’ mental health, with some limitations [16]. According to the social ecological model, mental health is affected by factors at multiple levels and interaction between factors. However, most studies explored factors at a single level or a single type of factors. For instance, studies focused on factors either at individual (psychological characteristics) [17, 18], or interpersonal (e.g., social support) [19–21], organizational (e.g., workplace violence) [22], or societal level (e.g., social status) [23, 24]. Therefore, these studies fail to offer a complete picture of factors at multiple levels and examine interactions between factors. Additionally, the majority of extant studies adopt quantitative design with standardized measurements, which may neglect the intricacies of personal experiences and the significance of context.

To fill aforementioned gap, our study is aimed to explore associated factors for mental health at all four socio-ecological levels and to understand the interactions between factors from the perspective of clinical nurses.

## Methods

### Study design

This study adopted a qualitative descriptive design with focus group interviews. Qualitative description design is widely used to gather insight from key informants about poorly understood healthcare questions [25, 26]. The design was considered appropriate because this study aimed to obtain a detailed description of participants’ perceived influencing factors of mental health. Focus group interviews were used for data collection to encourage the free exchange of information and to yield richer data and deeper insights into the topic.

### Setting

This study was conducted in a tertiary hospital in Changsha, Hunan Province, China. The hospital has 3000 nurses and 137 head nurses.

### Participants

This study included clinical nurses and head nurses who were employed by the hospital for one year or over. They were recruited, using both convenience and purposive sampling between April to May 2023. The study was advertised through the existing network of the authors. Potential participants were approached by the authors via WeChat with an explanatory statement. The explanatory statement included a brief introduction of the study and invited potential participants to contact the first author directly to arrange the interview time and venue. Purposive sampling was used to obtain maximum variation, within participants’ characteristics including gender, years of work experience, clinical work area, and having an administrative position or not.

### Data collection

We conducted seven focus groups (seven- eight participants in each group) in the meeting room of the hospital between April to May 2023. We introduced the purpose of the research and topics before conducting the group interview. The interview guide were developed based on the literature review, including following questions: (1) How about your mental health in daily work? (2) What are the factors influencing your mental health? (3) How does mental health affect your daily life? (4) When you felt down, what kind of coping strategies do you adopted? (5) What external factors (e.g., individual, interpersonal and environmental factors) are conducive to promoting your mental health? The interviews were conducted in Mandarin. The second author acted as a facilitator for focus groups, and she participated workshop in qualitative research as part of master course. The fourth author acted as a note taker who took field notes and observed the interaction within the groups. The duration of the focus group interviews ranged from 65 to 94 min (mean 81.5 min).

### Data analysis

Preliminary data collection and data analysis were conducted simultaneously, which enabled collection to cease on reaching data saturation. All audio recordings were transcribed in Mandarin using Xunfei software, and the accuracy was verified by the first, third, and fifth authors. Then, all the data were input entered into excel for analysis. Three authors (the second, eighth, and ninth authors) independently coded the transcripts line by line and then deliberated to form a preliminary coding framework. Constant comparative analysis ensured consistent coding across transcripts. They developed a preliminary coding framework after coding the first three transcripts, refining it iteratively with subsequent transcripts. This was repeated with further transcripts, and the subthemes were refined and reduced in number by grouping codes together. Following the development of the final coding framework, the remaining transcripts remained open to new additions if needed.

Final themes were constructed using an inductive process. The social ecological model was used to group themes. This model was used to connect the findings with the literature and conceptual framework. The social ecological model [27] is used to describe multiple factors affecting mental health and explore healthcare behaviors [28, 29], these factors grouped into four levels: intrapersonal, interpersonal, organizational and societal level. This model includes four levels: individual, interpersonal, organizational and societal. Individual level identifies biological, character traits and psychological factors. Interpersonal level examines communication and interaction with individuals in social networks. Organizational level contains resources obtained from organizations and through social interactions. Societal level focuses on factors that help create an atmosphere conducive to maintaining mental health.

### Rigour

The study’s rigor was established through meticulous attention to credibility, transferability, dependability, and confirmability [30]. Credibility was achieved by rigorously analyzing the data by the research team. Transferability was ensured by providing a comprehensive description of the study setting and detailed narratives of participant experiences. Additionally, dependability and confirmability were upheld through a meticulous audit of methodological decisions made by the research team throughout the study process.

## Results

### Participant’s characteristics

Fifty nurses were invited to participate in this study, and two declined the invitation; the remaining 48 nurses completed the interview. More female nurse participated in the study (*n* = 37) rather than male (*n* = 5). The participants’ social demographic characteristics are presented in Table 1.

**Table 1:** Demographics of participants (*n* = 48)

Number	Gender	Work experience	Clinical work area	Position
F1P1	Female	10	Intensive care unit	Nurse
F1P2	Male	5	Intensive care unit	Nurse
F1P3	Female	10	Intensive care unit	Nurse
F1P4	Female	1	Intensive care unit	Nurse
F1P5	Male	21	Intensive care unit	Nurse
F1P6	Female	7	Intensive care unit	Nurse
F1P7	Female	10	Intensive care unit	Nurse
F1P8	Female	17	Intensive care unit	Nurse
F2P1	Male	10	Operating room	Nurse
F2P2	Female	6	Operating room	Nurse
F2P3	Female	18	Operating room	Nurse
F2P4	Female	10	Operating room	Nurse
F2P5	Female	14	Operating room	Nurse
F2P6	Female	8	Operating room	Nurse
F3P1	Female	11	Ophthalmology and otorhinolaryngology	Nurse
F3P2	Female	5	Ophthalmology and otorhinolaryngology	Nurse
F3P3	Female	15	Ophthalmology and otorhinolaryngology	Nurse
F3P4	Female	5	Ophthalmology and otorhinolaryngology	Nurse
F3P5	Female	3	Ophthalmology and otorhinolaryngology	Nurse
F3P6	Female	12	Ophthalmology and otorhinolaryngology	Nurse
F3P7	Female	27	Ophthalmology and otorhinolaryngology	Nurse
F4P1	Male	8	Emergency	Nurse
F4P2	Female	8	Emergency	Nurse
F4P3	Female	17	Emergency	Nurse
F4P4	Female	11	Emergency	Nurse
F4P5	Female	10	Emergency	Nurse
F4P6	Male	3	Emergency	Nurse
F4P7	Female	31	Emergency	Nurse
F5P1	Female	3	Surgery	Nurse
F5P2	Female	12	Surgery	Nurse
F5P3	Female	11	Surgery	Nurse
F5P4	Female	7	Surgery	Nurse
F5P5	Female	26	Surgery	Nurse
F5P6	Female	12	Surgery	Nurse
F5P7	Female	10	Surgery	Nurse
F6P1	Female	5	Internal medicine	Nurse
F6P2	Female	10	Internal medicine	Nurse
F6P3	Female	9	Internal medicine	Nurse
F6P4	Female	10	Internal medicine	Nurse
F6P5	Female	30	Internal medicine	Nurse
F6P6	Female	15	Internal medicine	Nurse
F6P7	Female	13	Internal medicine	Nurse
F7P1	Female	22	Intensive care unit	Head nurse
F7P2	Female	19	Emergency	Head nurse
F7P3	Female	11	Surgery	Head nurse
F7P4	Male	6	Internal medicine	Head nurse
F7P5	Female	29	Internal medicine	Head nurse
F7P6	Female	27	Intensive care unit	Head nurse

### Main findings

**Fig. 1:**
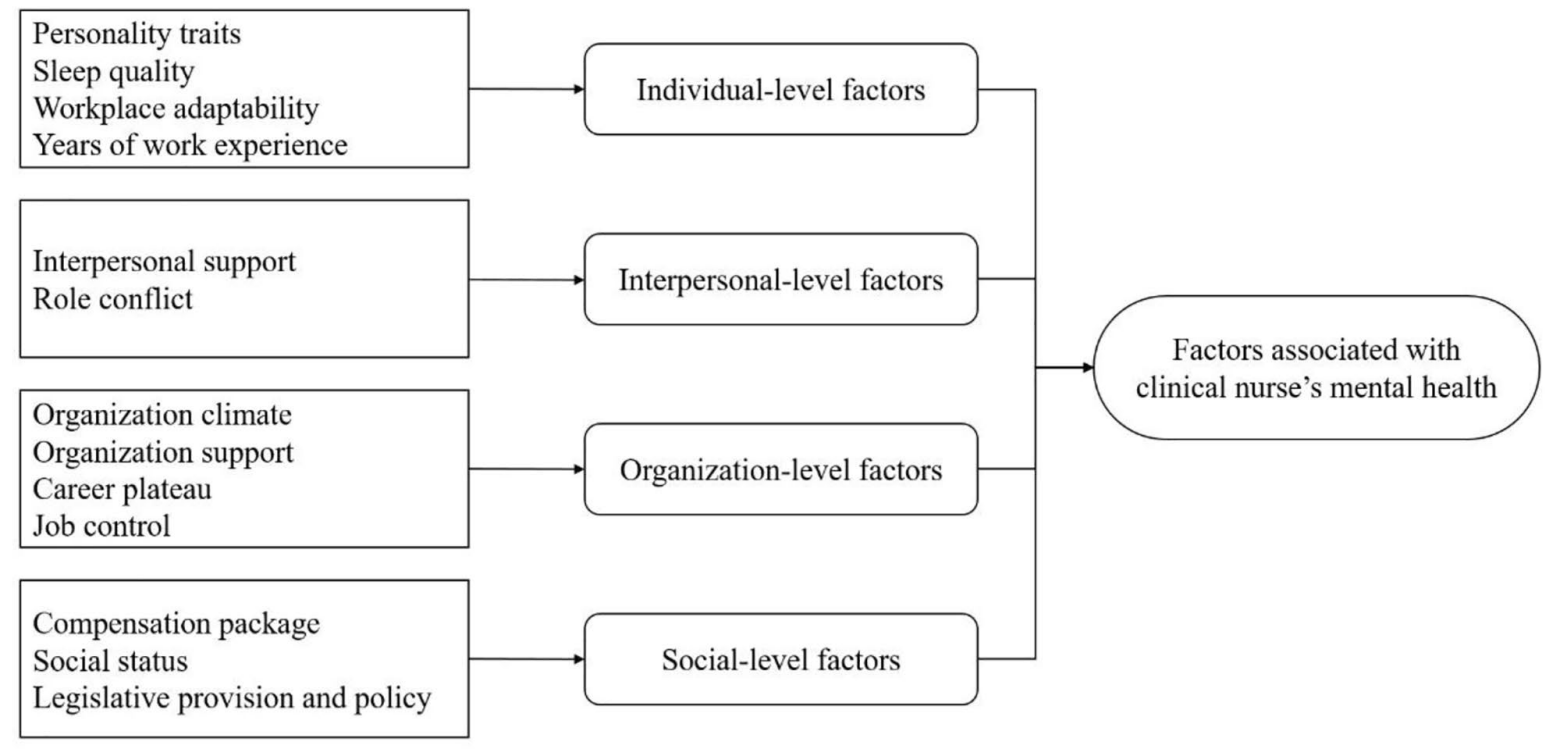
Factors associated with clinical nurse’s mental health

As shown shown in Fig. 1, factors associated with clinical nurses’ mental health were categorized four themes and 13 subthemes: (1) individual-level factors, (2) interpersonal-level factors, (3) organization-level factors, and (4) social-level factors.

### Individual-level factors

Participants reported that their mental health could be impacted by personality traits (i.e., optimistic/negative life outlook), quality of sleep, workplace adaptability, and years of work experience. Some participants mentioned that adaptability was important for them to manage emotional and practical daily challenges in the face of rapidly changing and unpredictable circumstances.

*When novice nurses take care of patients by themselves, they may experience increased stress, especially when patients’ condition changes suddenly during the night shift (F1P4).*

### Interpersonal-level factors

Participants perceived that interpersonal support and role conflict were associated with their mental health.

#### Interpersonal support

Our participants identified that interpersonal support was playing an important role in maintaining their mental health. They explained that talking to their families, friends, colleagues and supervisor were an effective way to relieve work stress.

*I sought to the person I trust the most (my family) and talked all the unpleasant things with them when I felt very stressed (F3P3).*

#### Role conflict

Participants mentioned that it was inevitable for them to experience role conflict (i.e., work-family conflict and work-school conflict) because of the demanding and challenging conditions of the job. They felt guilty when work pressures interfered with family responsibilities. Some participants identified that their emotional stress increased when their work interfered with their ability to meet the demands of their kids’ school. The demands of long study hours and early clinical hours caused stress among them and kept them from household responsibilities of cooking, cleaning, and spending time with children. Participants also felt that family support of their career choices helped their job performance.

.*my father was diagnosed with lung cancer two years ago. He was resuscitated many times during his treatment. However, I was always busy working at the fever outpatient department and couldn’t spend much time with him. I still feel sad…(F7P4)*.

### Organization-level factors

Participants perceived that their mental health was influenced by the following four organizational-level factors, including (1) organization climate, (2) organization support, (3) career plateau, and (4) job control.

#### Organization climate

In this study, organization climate included emotional climate and workplace incivility. Participants perceived the importance of the emotional climate due to the transmissive nature of emotional states. It was easy to be infected by the negative emotions of colleagues, so that the entire department can generate or maintain a negative emotional climate, vice versa.

*Some colleagues are always complaining, which affects others’ the mood (F3P4).*

Most participates identified it was common for them to experience workplace incivility which came from their nurses, physicians, supervisors and patients. They felt disrespected, threatened reprimanded, and emotionally abused, which evoke negative emotions, such as anxiety, depression, exhaustion.

#### Organization support

Participants perceived that organization support (i.e., instrumental and emotional support) were related to job satisfaction and mental health. Participants identified various forms of instrumental support, including physical environment, sufficient human resource, task assistance, training opportunities and flexibility in work schedule. The support helped them to perform job roles, which also carried emotional meanings. Emotional support included listening to work concerns, allowing to vent emotions, and providing words of encouragement. The support provided socioemotional resources, involving affection, sympathy, understanding, acceptance, and recognition.

.*we definitely don’t want our supervisor to scold us without getting the full picture (of the whole thing), and we really hope that supervisor investigate what really happened…(F7P2)*.

#### Career plateau

Our participants, especially seniors frequently mentioned the challenge of double career plateau which includes hierarchical plateau and content plateau. They felt frustrated and even hopeless when they were experiencing a permanent end in career advancement. Some participants perceived little opportunity for vertical improvement because of the flattened pyramid shape within the hospital. Some participants expressed the concern about future professional recession because they have limited opportunities to master new skills.

*Everyone think that our nurses don’t seem to have a future, especially the male nurses… only one or two nurses can really be head nurses (F2P1).*

#### Job control

Many participates complained that they lack of control over work time and tasks. They had to extend their work time without compensation, leading to work-family conflicts. They felt exhausted and disgusted when they were asked to attend training and meetings immediately after night shifts. Additionally, some participants got annoyed by research tasks because they were not interested in it, and some participants felt incompetent at it because they did not receive relevant training.

*we were asked to attend meetings and participate training and other activities after we finish our night shift. It’s really annoying (F7P5).*

### Social-level factors

Participants identified three social-level factors associated with the mental health, including (1) compensation package, (2) social status, and (3) legislative provision and policy.

#### Compensation package

Many participants were not satisfied with their compensation package. They indicated feelings of inadequate reward for their efforts and the level of responsibility, and unfairness of salary compared with doctors. Some participants felt unsafe because the institute did not buy pension insurance for them.

*I did not have pension insurance, I feel stressed (laughing)… I reckon that as long as our profession enjoys good welfare and incentives…People will regard nursing as a valuable profession…(F2P8)*.

#### Social status

Some participants perceived their social status as low, and it is common for them to receive discrimination from patients, relatives and doctors. Participants shared their experience of being viewed as servants by patients in the ward, which made them feel humiliated. They frankly voiced that their low social status, low salary and unsatisfactory professional image made them reluctant to recommend this career to others.

*…In the eyes of most people, our status, ,are indeed low, they (patients) look down on us as if we were just waiters (F7P7)…*.

#### Legislative provision and policy

Participants believe that legislative provision and supportive policy was an effective approach to improve social status and professional image.

*How do you advocate for the rights of nurses? I believe the legal aspect is more important…(F5P4)*.

## Discussion

To our knowledge, this is the first qualitative study which explored factors associated with mental health of clinical nurses by using socio-ecological model. The study advances the literature by emphasizing (1) the mental health is influenced by multi-level factors which include intrapersonal - (i.e., personality traits, quality of sleep, workplace adaptability, and years of work experience), interpersonal (i.e., interpersonal support and role conflict), organizational (i.e., organization climate, organization support, career plateau, and job control), and social-level factors (i.e., including compensation package, social status, and legislative provision and policy), (2) the interaction between factors, and (3) the reciprocal relationship between individuals’ mental health and their environments.

Consistent with the findings of previous research [31–35], our study found that nurses experience more work-to-family conflict than family-to-work conflict, leading to a feeling of stress and guilt. This may be because work and family life are mutually incompatible to some extent. Nurses experience high levels of physical, cognitive, and emotional demands due to the nature of the nursing profession. Meanwhile, most nurses are women, indicating a substantial number of dual-career or single-woman-headed households. They always are expected to take the primary responsibility for childcare and housework by themselves and society [36]. Therefore, they feel guilty when their work interferes with household duties and family responsibilities, or work detracts from quality time with their families. Notably, our study also found that organizational support (i.e., supportive working environment and flexibility in work schedule) and family support systems could help to mitigate work-family conflict. Consistently, organizational support has been identified as a valuable resource for fostering positive work attitudes and alleviating depressive symptoms [37, 38].

Our study recognized the occurrence of double career plateau in nursing. This is because hierarchical and content plateau are closely connected. For example, the hierarchical plateau could lead to the content plateau. Nurse staff are more like to decrease their effort and consciously avoid holding more responsibilities due to the absence of promotion opportunities. Vice versa, nurse staff who are unable to expand their job expertise have limited opportunity for promotion. Notable, our study found that some nursing staff have initiated strategies to manage career plateau by improving academic qualifications. This finding was supported by previous evidence showing that more and more nurses are pursuing master’s and doctorates degrees [39]. Therefore, those nurses are more likely to experience role conflict and have compromised mental health [40]. Because they must navigate the added role of a student in addition to their professional career and family responsibilities within limited time and energy [41]. The career plateau not only leads to mental health problems (e.g.,depression, psychological stress, and burnout) but also exerts adverse effects on physical health. These effects manifest as irritability, outbursts, deteriorating service attitudes, confrontations with managers [42]. Nursing organizations and managers can address career plateau by providing more opportunities for advancement in nursing positions and titles and by establishing multi-dimensional career advancement pathways. For instance, implementing hierarchical management for nurses [43] can diversify career opportunities, motivate them, and ease the sense of professional stagnation, thereby alleviating mental health issues linked to career plateaus.

Our study found that nurses experience workplace uncivil acts from various sources, involving other nurses, physicians, supervisors, patients, and visitors. Consistently, evidence indicated that 65.7 − 90.4% of nurses were exposed to some degree of incivility. Previous studies have examined how this destructive behaviour affects organizational and individual outcomes, and which factors influence it [44–48]. Workplace incivility could cause emotional distress and productivity losses in nurses. This situation may be detrimental to patient safety and satisfaction. These negative outcomes could leads to financial strain on healthcare organizations [49]. Uncivil interactions within the healthcare team could be triggered by organizational and interpersonal factors, such as lack of support, heavy workload, inadequate personnel, and long working shifts. Particularly, these interactions negatively affect nurses who are the backbone of the team. Similarly, these factors were identified as risk factors of mental health of nurses in our study. We also found that support from other supervisors and coworkers could create healthy work environment, which is associated with improved mental health of nurses.

### Strengths and limitations

A strength of this study was the use of the social ecological model as a theoretical framework. Contributory factors identified within each level of the framework were discussed by participants. This highlights that interventions developed around these contributory factors have the potential to improve clinical nurses’ mental health.

This study only recruited clinical nurses in one tertiary hospital, which may limits its generalizability. Our participants were recruited through the existing network of the author team, which may lead to selection bias.

## Conclusion

This groundbreaking study has utilized the socio-ecological model to illuminate the intricate web of factors influencing the mental health of clinical nurses. The findings underscore the need for holistic interventions that address not only intrapersonal and interpersonal factors but also organizational and social-level factors to promote nurses’ well-being. By acknowledging the complexities of the nursing profession, healthcare organizations, managers, and policymakers can take proactive steps to create supportive environments, foster career development, and mitigate the adverse effects of workplace incivility. Ultimately, these efforts hold the promise of enhancing the mental health and overall job satisfaction of clinical nurses, which in turn contributes to improved patient care and healthcare system performance.

## Data Availability

The datasets used and/or analysed during the current study are available from the corresponding author on reasonable request.
